# Probing entanglement in a 2D hard-core Bose–Hubbard lattice

**DOI:** 10.1038/s41586-024-07325-z

**Published:** 2024-04-24

**Authors:** Amir H. Karamlou, Ilan T. Rosen, Sarah E. Muschinske, Cora N. Barrett, Agustin Di Paolo, Leon Ding, Patrick M. Harrington, Max Hays, Rabindra Das, David K. Kim, Bethany M. Niedzielski, Meghan Schuldt, Kyle Serniak, Mollie E. Schwartz, Jonilyn L. Yoder, Simon Gustavsson, Yariv Yanay, Jeffrey A. Grover, William D. Oliver

**Affiliations:** 1 Research Laboratory of Electronics, Massachusetts Institute of Technology, Cambridge, MA USA; 2 Department of Electrical Engineering and Computer Science, Massachusetts Institute of Technology, Cambridge, MA USA; 3 Department of Physics, Wellesley College, Wellesley, MA USA; 4 Department of Physics, Massachusetts Institute of Technology, Cambridge, MA USA; 5 MIT Lincoln Laboratory, Lexington, MA USA; 6 Laboratory for Physical Sciences, College Park, MD USA; 7 Present Address: Google Quantum AI, Santa Barbara, CA USA

**Keywords:** Quantum simulation, Quantum information

## Abstract

Entanglement and its propagation are central to understanding many physical properties of quantum systems^1–3^. Notably, within closed quantum many-body systems, entanglement is believed to yield emergent thermodynamic behaviour^4–7^. However, a universal understanding remains challenging owing to the non-integrability and computational intractability of most large-scale quantum systems. Quantum hardware platforms provide a means to study the formation and scaling of entanglement in interacting many-body systems^8–14^. Here we use a controllable 4 × 4 array of superconducting qubits to emulate a 2D hard-core Bose–Hubbard (HCBH) lattice. We generate superposition states by simultaneously driving all lattice sites and extract correlation lengths and entanglement entropy across its many-body energy spectrum. We observe volume-law entanglement scaling for states at the centre of the spectrum and a crossover to the onset of area-law scaling near its edges.

## Abstract (web summary)

By emulating a 2D hard-core Bose–Hubbard lattice using a controllable 4 × 4 array of superconducting qubits, volume-law entanglement scaling as well as area-law scaling at different locations in the energy spectrum are observed.

## Main

Entanglement is a uniquely quantum property that underpins descriptions of interacting quantum systems as statistical ensembles^4–7^. Within closed many-body quantum systems, entanglement among constituent subsystems introduces uncertainty to their individual states, even when the full system is in a pure state^1,15^. For this reason, entropy measures are commonly used to quantify quantum entanglement in many-body systems and have been directly probed in different platforms^8–14^. The study of entanglement in interacting many-body quantum systems is central to the understanding of a range of physical phenomena in condensed-matter systems^1^, quantum gravity^2,3^ and quantum circuits^16^. The scaling of the entanglement entropy with subsystem size provides insight into classifying phases of quantum matter^17–19^ and the feasibility of numerically simulating their dynamics^15^.

**Fig. 1:**
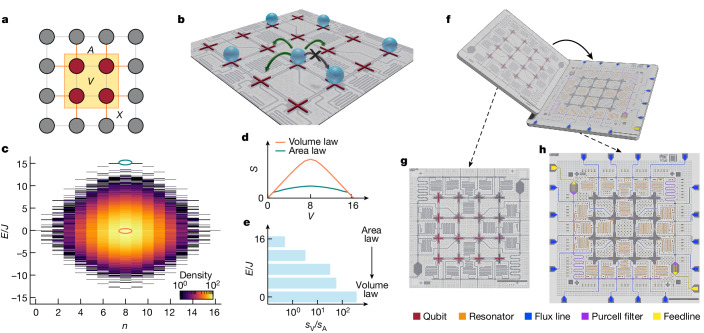
Experimental concept. **a**, Schematic for an example subsystem *X* of four qubits within a 16-qubit lattice. The subsystem has a volume of 4 (maroon sites) and an area of 8 (orange lines). **b**, 2D HCBH lattice emulated by the superconducting quantum circuit. Each site can be occupied by, at most, a single particle. **c**, Energy *E* spectrum of the HCBH lattice emulated by our device, shown in the rotating frame resonant with the lattice sites. The energy spectrum is partitioned into distinct sectors defined by the total particle number *n*. **d**, Scaling of the entanglement entropy *S* with subsystem volume *V* for an eigenstate at the centre of the energy spectrum (orange line, corresponding to the energy eigenstate highlighted by the orange oval in **c**) and an eigenstate at the edge of the energy spectrum (teal line, corresponding to the energy eigenstate highlighted by the teal oval in **c**). **e**, Change in the entanglement behaviour, quantified by the geometric entropy ratio *s*_V_/*s*_A_, for states with *n* = 8. **f**, Schematic for the flip-chip sample consisting of 16 superconducting qubits. **g**,**h**, Optical images of the qubit tier (**g**) and the interposer tier (**h**) are illustrated with the qubits and the different signal lines false-coloured. Scale bars, 1 mm.

In a closed system, the bipartite entanglement entropy of a subsystem quantifies the amount of entanglement between the subsystem and the remainder of the system. For certain many-body states, such as the ground state of 1D local Hamiltonians^20^, the entanglement entropy is proportional to the boundary between a subsystem and the remaining system; this boundary is referred to as the area of a subsystem. Such states are said to have area-law entanglement scaling^15^. For other states, entanglement entropy increases proportionally to the bulk size (volume) of a subsystem, a behaviour referred to as volume-law scaling. To characterize the entanglement scaling in an interacting lattice, we consider the area and volume entanglement entropy per lattice site, represented by *s*_A_ and *s*_V_, respectively. Disregarding logarithmic corrections, which are theoretically expected in certain contexts^21,22^, the entanglement entropy *S*(*ρ*_*X*_) of a subsystem *X* can be expressed using the ansatz

1 $$S({\rho }_{X})={s}_{{\rm{A}}}{A}_{X}\,+{s}_{{\rm{V}}}{V}_{X},$$

in which *A*_*X*_ is the subsystem area and *V*_*X*_ is the subsystem volume (see Fig. 1a for an example). The ratio of volume to area entropy per site, *s*_V_/*s*_A_, quantifies the extent to which that state obeys area-law or volume-law entanglement scaling^23^. Quantum states with area-law entanglement scaling have local correlations, whereas systems obeying volume-law entanglement scaling contain correlations that extend throughout the system and are therefore more challenging to study using classical numerical methods^24,25^.

In this work, we study the entanglement scaling of states residing in different energetic regions of a 2D HCBH lattice. The Bose–Hubbard model is particle-number conserving, allowing its energy spectrum to be partitioned into sectors with definite particle number *n*. The ‘hard-core’ condition arises from strong on-site particle–particle interactions that mandate that each lattice site may be occupied by at most a single particle (Fig. 1b). In 2D, the HCBH model is non-integrable and may exhibit eigenstate thermalization^5,26,27^ and quantum information scrambling^28^. Highly excited many-body states—states residing near the centre of the energy spectrum (energy *E* ≈ 0; orange oval in Fig. 1c)—are expected to exhibit volume-law scaling of the entanglement entropy, following the Page curve^2^ (orange line in Fig. 1d). For subsystems smaller than half the size of the system, entropy grows linearly in subsystem size. Entropy is maximized when the subsystem comprises half of the entire system and decreases as the subsystem size is further increased. By contrast, the entanglement entropy of states residing near the edges of the HCBH energy spectrum does not follow the Page curve but instead grows less rapidly with subsystem volume (exemplified by the teal line in Fig. 1d showing the entropy of the eigenstate highlighted by a teal oval in Fig. 1c). For states with intermediate energies, a crossover is expected from area-law scaling at the edges of the energy spectrum to volume-law scaling at its centre^22,23^ (Fig. 1e). Although Fig. 1e illustrates the crossover for the *n* = 8 particle-number manifold, the crossover similarly occurs in other manifolds (see Methods).

The crossover from area-law to volume-law entanglement scaling can be observed by studying the exact eigenstates of the HCBH model. However, preparing a specific eigenstate generally requires a deep quantum circuit^29^ or a slow adiabatic evolution^30^. Alternatively, we can explore the behaviour of the entanglement entropy by preparing superpositions of eigenstates across numerous particle-number manifolds^23^. Here we prepare such superposition states by simultaneously and weakly driving 16 superconducting qubits arranged in a 4 × 4 lattice. By varying the detuning of the drive frequency from the lattice frequency, we generate superposition states occupying different regions of the HCBH energy spectrum. Measurements of correlation lengths and entanglement entropies indicate volume-law entanglement scaling for states prepared at the centre of the spectrum and a crossover to the onset of area-law entanglement scaling for states prepared at the edges.

## Experimental system

**Fig. 2:**
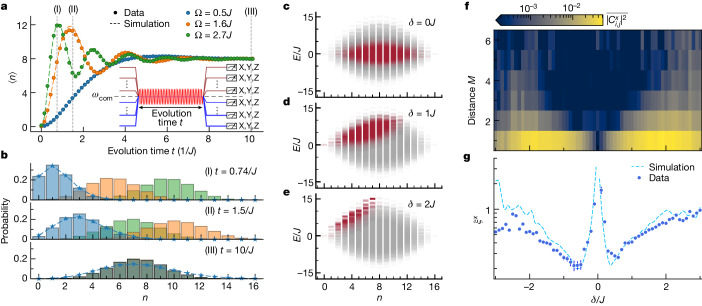
Coherent-like state preparation. **a**, Total number of particles ⟨*n*⟩ in the uniform lattice while driving the system on resonance for time *t*. Simulations do not include decoherence. The lattice reaches half-filling at equilibrium. The experiments are executed using the pulse sequence shown in the inset. **b**, Probability of measuring different numbers of excitations in the lattice at three different times. The blue stars are from a Poisson fit to the excitation-number distribution for Ω = *J*/2, with the dashed lines as guides to the eye. **c**–**e**, Simulated overlap of the prepared coherent-like state in steady state (*t* = 10/*J*) with drive strength Ω = *J*/2 and drive detuning *δ* = 0*J* (**c**), *δ* = 1*J* (**d**) and *δ* = 2*J* (**e**) with the HCBH energy eigenstates. The different shades of red indicate the magnitude of the overlap between the prepared superposition states and energy eigenstates. Note that the spectra are shown in the rotating frame of the lattice sites and not of the drive. **f**, Average two-point correlator squared along the *x* basis, $\overline{| {C}_{i,j}^{x}{| }^{2}}$, between qubit pairs at distance *M* for drive duration *t* = 10/*J*, strength Ω = *J*/2 and detuning *δ* from the lattice frequency. **g**, Correlation length *ξ*^*x*^ extracted using the two-point correlators at different values of *δ*.

Superconducting quantum processors provide strong qubit–qubit interactions at rates exceeding individual qubit decoherence rates, making them a platform that is well suited for emulating many-body quantum systems^28,30–36^. In this experiment, we use a 2D lattice of 16 capacitively coupled superconducting transmon qubits^37^, fabricated in a flip-chip geometry^38^ (Fig. 1f,g; see details in Methods). The circuit emulates the 2D Bose–Hubbard model, described by the Hamiltonian

2 $${\widehat{H}}_{{\rm{BH}}}/\hbar =\sum _{i}{{\epsilon }}_{i}{\widehat{n}}_{i}+\sum _{i}\frac{{U}_{i}}{2}{\widehat{n}}_{i}\left({\widehat{n}}_{i}-1\right)+\sum _{\langle i,j\rangle }{J}_{ij}{\widehat{a}}_{i}^{\dagger }{\widehat{a}}_{j},$$

in which ${\widehat{a}}_{i}^{\dagger }$ (${\widehat{a}}_{i}$) is the creation (annihilation) operator for qubit excitations at site *i*, ${\widehat{n}}_{i}={\widehat{a}}_{i}^{\dagger }{\widehat{a}}_{i}$ is the respective excitation number operator and qubit excitations correspond to particles in the Bose–Hubbard lattice. The first term represents the site energies *ϵ*_*i*_ = *ω*_*i*_ − *ω*_r_, in which the qubit transition frequencies *ω*_*i*_ are written in a frame rotating at frequency *ω*_r_. The second term describes on-site interactions arising from the qubit anharmonicities *U*_*i*_, with an average strength *U*/2π = −218(6) MHz. The final term of the Hamiltonian describes particle-exchange interactions of strengths *J*_*ij*_ between neighbouring lattice sites with an average strength of *J*/2π = 5.9(0.4) MHz at qubit frequency *ω*/2π = 4.5 GHz. Particle exchange during state preparation and readout is prevented by detuning the qubits to different frequencies (inset in Fig. 2a). Our system features site-resolved, multiplexed single-shot dispersive qubit readout^39^ with an average qubit state assignment fidelity of 93%, which—together with site-selective control pulses—allows us to perform simultaneous tomographic measurements of the qubit states.

In our system, on-site interactions are much stronger than exchange interactions, *J* ≪ |*U*|. By restricting each site to two levels and mapping the bosonic operators to qubit operators, we transform the Hamiltonian in equation (2) to the hard-core limit

3 $${\widehat{H}}_{{\rm{HCBH}}}/\hbar =\sum _{\langle i,j\rangle }{J}_{ij}{\widehat{\sigma }}_{i}^{+}{\widehat{\sigma }}_{j}^{-}-\sum _{i}\frac{{{\epsilon }}_{i}}{2}{\widehat{\sigma }}_{i}^{z},$$

in which ${\widehat{\sigma }}_{i}^{+}$ (${\widehat{\sigma }}_{i}^{-}$) is the raising (lowering) operator for a qubit at site *i* and ${\widehat{\sigma }}_{i}^{z}$ is the Pauli Z operator. The energy relaxation rate Γ_1_ and dephasing rate Γ_*ϕ*_ are small compared with the particle-exchange rate, with Γ_1_ ≈ 10^−3^*J* and Γ_*ϕ*_ ≈ 10^−2^*J*, allowing us to prepare many-body states and probe their time dynamics faithfully.

## Coherent-like states

We generate a superposition of many eigenstates of the lattice, which we refer to as a coherent-like state, by simultaneously driving all qubits through a common control line. Applied to the lattice initialized with no excitations, the drive acts as a displacement operation of the Fock basis defined by the number of excitations in the lattice^23^, hence, it will result in a state analogous to coherent states of light. Selecting the rotating-frame frequency *ω*_r_ to be the frequency of the drive, the Hamiltonian of the driven lattice is

4 $$\widehat{H}/\hbar =\sum _{\langle i,j\rangle }{J}_{ij}{\widehat{\sigma }}_{i}^{+}{\widehat{\sigma }}_{j}^{-}+\frac{\delta }{2}\sum _{i}{\widehat{\sigma }}_{i}^{z}+\Omega \sum _{j}({\alpha }_{j}{\widehat{\sigma }}_{j}^{-}\,+\,\text{h.c.}),$$

in which *δ* = *ω*_r_ − *ω*_com_ is the detuning between the drive and the qubit frequencies (all qubits are biased on resonance at *ω*_com_). The drive strength Ω can be tuned by varying the amplitude of the applied drive pulse. The common drive couples independently to each qubit with a complex coefficient *α*_*j*_ that depends on the geometric circuit parameters of the lattice.

We study the time dynamics of the average number of excitations ⟨*n*⟩ in the lattice under a resonant drive (*δ* = 0) in Fig. 2a. The driven lattice reaches a steady state of half-filling, with an average particle number ⟨*n*⟩ = 8, after driving the lattice for time *t* ≈ 10/*J*. Once in steady state, the drive adds and removes excitations coherently from the system at the same rate. Hamiltonian parameters *J*_*ij*_ and *α*_*j*_ were characterized through a procedure described in Section 4 of the Supplementary Information and the excellent agreement of numerical simulations of time evolution under equation (4) with experimental data confirms their accuracy. In Fig. 2b, we report the discrete probability distribution of measuring a different number of excitations in the lattice at three different times. The probability of a particular excitation number approximately follows a Poisson distribution for a weak drive Ω = *J*/2 (blue stars with dashed lines as guides to the eye), indicating that the quantum state is in a coherent-like superposition of excitation-number states.

The coherent-like state comprises a swath of the HCBH energy spectrum that depends on the drive detuning. Driving the lattice on resonance prepares a coherent-like state at the centre of the HCBH energy spectrum (Fig. 2c). By varying the drive detuning, we can generate states that are a superposition of the eigenstates closer to the edge of the energy band (Fig. 2d,e). The standard deviation in the energy of the state depends on the strength of the drive: a stronger drive increases the bandwidth of populated energy eigenstates within each particle-number subspace. Therefore, to probe the entanglement properties across the lattice energy spectrum, we choose a relatively weak drive with strength Ω = *J*/2 for the rest of the main text. We choose a drive duration *t* = 10/*J*, which is short compared with the timescale of decoherence, yet long enough to allow the coherent-like state to reach its steady-state distribution (which occurs after roughly *t* = 6/*J* according to simulations shown in Section 14 of the Supplementary Information).

## Correlation lengths

A system of interacting particles exhibits correlations between its constituent subsystems. The HCBH Hamiltonian is equivalent to an XY Hamiltonian, in which quantum order is reflected by the transverse correlations^40^. To quantify transverse correlations, for states generated with detuning *δ*, we measure the two-point correlators ${C}_{i,j}^{x}\equiv \langle {\widehat{\sigma }}_{i}^{x}{\widehat{\sigma }}_{j}^{x}\rangle -\langle {\widehat{\sigma }}_{i}^{x}\rangle \langle {\widehat{\sigma }}_{j}^{x}\rangle$ between different qubit pairs. In Fig. 2f, we show the magnitude-squared two-point correlator values $\overline{| {C}_{i,j}^{x}{| }^{2}}$, averaged over qubits at the same Manhattan distance *M*. When *δ*/*J* is small, generating a superposition state occupying the centre of the energy band, $\overline{| {C}_{i,j}^{x}{| }^{2}}$ becomes small for all *M*, matching the expectation for states with volume-law entanglement scaling. The two-point correlator has an upper bound set by the mutual information between the sites *I*(*i*: *j*) = *S*(*ρ*_*i*_) + *S*(*ρ*_*j*_) − *S*(*ρ*_*ij*_). In a volume-law state, the entropy of small subsystems is equal to their volume^2^, hence we expect that the mutual information—and, in turn, the correlator between any qubit pair—will vanish.

Using the two-point correlators, we extract the correlation length *ξ*^*x*^ by fitting $| {C}_{i,j}^{x}{| }^{2}\propto \exp \left(-M/{\xi }^{x}\right)$ (Fig. 2g). The correlation length quantifies the dependence of correlations on the distance between the subsystems within our system. As we increase the magnitude of the drive detuning *δ*, skewing the superposition state to the edge of the energy spectrum, we generally find that the correlation length increases. The states prepared with −*J*/2 ≲ *δ* ≲ *J*/2, however, follow the opposite trend, and the extracted correlation length diverges around *δ* = 0. In this regime in which the coherent-like state occupies the centre of the energy spectrum, the two-point correlators asymptotically reach zero, so the extracted two-point correlation length loses meaning. We attribute the slight asymmetry of the observed correlation lengths about *δ* = 0 to a slight offset of the energy spectrum of our lattice towards positive energies owing to next-nearest-neighbour exchange interactions (see Section 5 of the Supplementary Information).

## Entanglement scaling behaviour

**Fig. 3:**
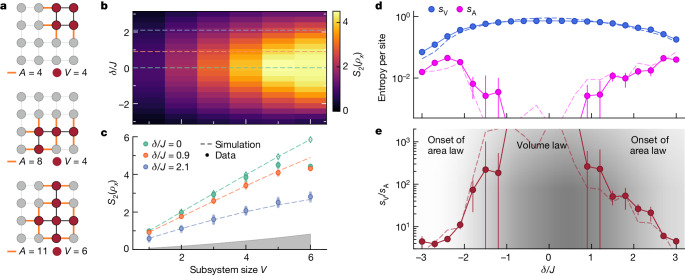
Entanglement scaling behaviour. **a**, Examples of subsystems measured in our lattice. **b**, The average subsystem entanglement entropy *S*_2_(*ρ*_*X*_) for a subsystem with volume *V* for a state prepared with drive time *t* = 10/*J*, strength Ω = *J*/2 and detuning *δ* from the lattice frequency. **c**, Subsystem entanglement entropy *S*_2_(*ρ*_*X*_) along the dashed lines in panel **b** for detunings *δ*/*J* = 0 (green), 0.9 (orange) and 2.1 (blue). Solid circles are experimental data averaged across all subsystems of each volume, with data for individual subsystems indicated by smaller, transparent circles. For *δ*/*J* = 0 (green), open diamonds are Monte Carlo simulations of 20,000 samples indicating that deviations at subsystem sizes 5 and 6 arise partially from insufficient sampling. The grey region indicates the estimated classical entropy from dephasing (see Supplementary Information for details). **d**, The volume entanglement entropy, *s*_V_, and area entanglement entropy, *s*_A_, per site extracted using 163 different subsystems of various volumes and areas for states prepared with drive detuning *δ*. The error bars indicate ±1 standard error of the fit parameter. **e**, The geometric entropy ratio *s*_V_/*s*_A_ is used for quantifying the behaviour of entanglement. States prepared with *δ* = 0 exhibit a strong volume-law scaling and the states prepared with larger drive detuning values show a weaker volume-law scaling with an increasing area-law scaling. Dashed lines in **c**–**e** indicate results from numerical simulation.

To study the entanglement scaling of the coherent-like states, we reconstruct the density matrices of 163 unique subsystems using a complete set of tomography measurements. The measured subsystems contain up to six qubits (see Fig. 3a for examples). We quantify the entanglement entropy between subsystem *X* and the remaining system through the second Rényi entropy^41^

5 $${S}_{2}\left({\rho }_{X}\right)=-{\log }_{2}{\rm{Tr}}\left({\rho }_{X}^{2}\right),$$

in which *ρ*_*X*_ is the reduced density matrix describing a subsystem *X* of the quantum system *ρ*.

We first study the scaling of the entanglement entropy with the subsystem volume *V*_*X*_, defined as the number of qubits within the subsystem, for states prepared with different drive detunings *δ* (Fig. 3b). For states prepared with *δ* = 0, we observe nearly maximal scaling of the entropy with subsystem size *S*_2_(*ρ*_*X*_) ≈ *V*_*X*_, whereas with increasing *δ*, the entropy grows more slowly with subsystem size (Fig. 3c).

There is excellent agreement between the expected and the measured entropy for subsystems of volume ≤4. Yet there is a discrepancy for the largest subsystems for which the coherent-like states are prepared near the centre of the spectrum (see Fig. 3c). The discrepancy arises, in large part, from having a finite number of measurement samples when reconstructing subsystem density matrices. Tomographic reconstruction of a subsystem state requires measuring Pauli strings. When the system has volume-law entanglement, the measurement outcome distributions become more uniform, increasing sensitivity to sampling errors, especially for larger subsystems. Therefore, the reconstructed density matrices of the largest subsystems have less entropy than the actual states, as seen for *V* = 5 and 6 in Fig. 3c. Monte Carlo simulations of measurement outcomes confirm that a more accurate reconstruction of highly entangled states follows from larger numbers of measurement samples of each Pauli string (see Methods). From our experimental data, we can extract 2,000 × 3^6−*V*^ measurement samples for each Pauli string describing a subsystem of volume *V*—sufficient for *V* = 1–4 but less so for *V* = 5 and 6. We could have obtained better agreement for *V* = 5 and 6 if we had used 20,000 samples (open diamonds in Fig. 3c), which would have been straightforward to implement experimentally.

We next determine the scaling of entanglement entropy with subsystem volume *s*_V_ and area *s*_A_ as per equation (1). Using the Rényi entropies of the density matrices reconstructed from experimental data, we extract *s*_V_ and *s*_A_ by measuring the rate of change of *S*_2_(*ρ*_*X*_) with *V*_*X*_ and the subsystem area *A*_*X*_, respectively, with the other held fixed. Here *A*_*X*_ is defined as the number of nearest-neighbour bonds intersecting the boundary of the subsystem *X*. The linear fitting procedure used to determine *s*_V_ and *s*_A_ is detailed in Section 10 of the Supplementary Information. In Fig. 3d, we observe that, as the magnitude of *δ* becomes larger, *s*_V_ decreases and *s*_A_ increases. Although extraction of *s*_V_ is reliable at all drive detunings, at small drive detuning values (−*J* < *δ* < *J*), the entanglement entropy does not exhibit a notable dependence on the area of the subsystem in our finite lattice, hence we are not able to reliably fit *s*_A_. By considering the geometric entropy ratio *s*_V_/*s*_A_, we observe a change in the behaviour of entanglement entropy within our system (Fig. 3e). The states prepared at the centre of the energy spectrum with *δ* = 0 exhibit a strong volume-law scaling and the states prepared closer to the edge of the energy spectrum show weaker volume-law scaling and increasing area-law scaling.

The entanglement spectrum, obtained through Schmidt decomposition, further quantifies the structure of entanglement across a bipartition of a closed quantum system^17^. The quantum state |*ψ*⟩ can be represented as a sum of product states of the orthonormal Schmidt bases |*k*_*X*_⟩ for a given subsystem *X* and $\left|{k}_{\bar{X}}\right\rangle$ for the remaining lattice^42^:

6 $$\left|\psi \right\rangle =\sum _{k}{\lambda }_{k}\left|{k}_{X}\right\rangle \left|{k}_{\bar{X}}\right\rangle ,$$

in which positive scalars *λ*_*k*_ are the Schmidt coefficients with ${\sum }_{k}{\lambda }_{k}^{2}=1$. The Schmidt coefficients form the entanglement spectrum and provide a proxy for the degree of entanglement between the two subsystems. For a subsystem *X* maximally entangled with the remaining lattice, all of the Schmidt coefficients will have an equal value ${\lambda }_{k}=1/\sqrt{{2}^{{V}_{X}}}$, in which *V*_*X*_ is the volume of *X* (assuming that ${V}_{X} < {V}_{\bar{X}}$).

**Fig. 4:**
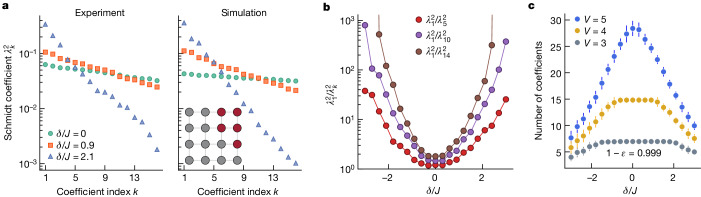
Schmidt coefficients scaling across the spectrum. **a**, The Schmidt coefficients for the decomposition of a five-qubit subsystem (highlighted with maroon colour in the inset) and the remaining lattice. Coherent-like states are prepared with different drive detunings *δ*/*J* = 0, 0.9 and 2.1. The coefficients in the left panel are calculated from experimental data and agree well with the simulated coefficients in the right panel. **b**, The ratio of the largest and the *k*th largest Schmidt coefficient ${\lambda }_{1}^{2}/{\lambda }_{k}^{2}$, for *k* = 5, 10 and 14, of coherent-like states prepared with different drive detunings *δ* in the experiment. **c**, The number of Schmidt coefficients required to represent the bipartition of the lattice with subsystem volume *V* = 3, 4 and 5 with accuracy 1 − *ϵ* = 0.999. Each data point is averaged over all the measured subsystems of the same size, with the error bars indicating ±1 standard deviation.

We obtain the entanglement spectrum for a bipartition of our lattice by diagonalizing the measured density matrix of a subsystem. In Fig. 4a, we study the entanglement formed within states prepared across the energy spectrum. We report the first 16 Schmidt coefficients squared ${\lambda }_{k}^{2}$, in decreasing order, for a subsystem highlighted in maroon and the remaining lattice. We observe that, for states obeying volume-law entanglement scaling at the centre of the spectrum, the variation in coefficient magnitudes is small compared with states closer to the edge of the spectrum, in close agreement with numerical simulation. To quantify this difference, in Fig. 4b, we show the ratio of the largest and the *k*th largest Schmidt coefficient, ${\lambda }_{1}^{2}/{\lambda }_{k}^{2}$, for *k* = 5, 10 and 14, of coherent-like states prepared with different drive detunings *δ*. We observe that a small number of Schmidt states contain nearly all the weight of the decomposition for the area-law-like states, whereas, for the volume-law states, the Schmidt coefficients are roughly equal. This variation signals a change in the extent of the entanglement distribution across the system.

In Fig. 4c, we report the number of coefficients required to approximate the state of the lattice bipartition with accuracy 1 − *ϵ* = 0.999 (see Section 12 of the Supplementary Information) for subsystems with volume *V* = 3, 4 and 5. We find that the states at the edge of the energy spectrum can be accurately represented with fewer coefficients, whereas the number of coefficients needed to approximate states at the centre of the band approaches the dimension of the Hilbert space of the subsystem, 2^*V*^.

## Conclusion

In this work, we study the entanglement scaling properties of the 2D HCBH model, emulated using a 16-qubit superconducting quantum processor. By simultaneously driving all qubits, we generate coherent-like superposition states that preferentially incorporate eigenstates from regions of the many-body energy spectrum that we tune between the centre and the edges. We probe the transverse quantum correlation lengths and the entanglement scaling behaviour of the superposition states. We observe a crossover from volume-law scaling of entanglement entropy near the centre of the band, coinciding with vanishing two-point correlators, to the onset of area-law entropy scaling at the edges of the energy band, accompanied by finite-range correlations.

The coherent-like superposition states comprise eigenstates across a swath of the HCBH energy spectrum. We can decrease the spectral width of these states by reducing the drive strength. However, the system under a weaker drive requires a longer evolution time to reach steady state. Improved processor coherence enables the creation of narrower-width states, providing a finer resolution for studying the area-to-volume-law crossover.

Although an analytical relation between entanglement and thermodynamic entropy exists for integrable systems^43^, the emergence of thermodynamic behaviour in non-integrable systems is less well understood^4–6,44,45^. In recent years, random-circuit protocols using digital quantum circuits have been applied to study this emergence^46^. The protocol introduced in this work is an analogue (that is, continuous-time evolution) counterpart to random-circuit experiments, one that is also capable of generating highly entangled states.

Our area-to-volume-law transition protocol applies to larger system sizes, despite the exponential time complexity of implementing state tomography, because we need not concomitantly increase the subsystem size (see simulations in Section 11 of the Supplementary Information). A subsystem with *V* qubits can probe entanglement correlations up to a depth of 2*V* qubits^47^. Therefore, a fixed subsystem volume and simultaneous readout ensure an essentially constant runtime and measure up to a fixed entanglement depth, even as the overall system size increases. Increasing the subsystem size does increase the accessible entanglement depth, but it comes with exponential cost. Therefore, our approach enables us to study emergent thermalization up to constant entanglement depth, even as we enter classically intractable regimes. The measurement time and depth are ultimately set by the subsystem size we are willing to accommodate.

Finally, the structure of entanglement within a quantum system determines the effective degrees of freedom required to accurately simulate the quantum states. Area-law states can generally be numerically simulated efficiently using tensor network methods^15,24,25^, whereas the computational complexity of classically simulating volume-law states scales exponentially with system size. It is the latter complexity that underpins the promise of quantum computational advantage. In this work, we demonstrated a hardware-efficient means to determine—to constant depth—the entanglement scaling and, thereby, the computational complexity of programs executed on quantum processors.

Note that, during the preparation of this manuscript, we became aware of related studies in a 1D trapped-ion simulator^48^.

## Methods

### Experimental setup

The experiment is performed in a dilution refrigerator at a base temperature of 20 mK. We study a superconducting processor with 16 transmon qubits arranged in a 4 × 4 square grid. The superconducting processor has aluminium circuit elements deposited on silicon substrates and is fabricated using a flip-chip process^38^, as depicted in Fig. 1f. The qubits are located on a qubit tier (Fig. 1g) and the readout and control lines are located on a separate interposer tier (Fig. 1h; see Supplementary Information for further device details).

Each transmon qubit in the superconducting processor represents one site in the Bose–Hubbard Hamiltonian. The site energy *ω*_*i*_ is given by the transition frequency from the ground state to the first excited state of the qubit and can be controlled with an error of less than 300 kHz (5 × 10^−2^*J*) (ref. ^49^). The on-site interaction *U*_*i*_ arises from the anharmonicity of transmon qubit *i*, representing the energy cost for two particles to occupy the same site. For transmon qubits, the energy cost is negative. The particle-exchange interaction between neighbouring lattice sites is realized by capacitively coupling adjacent qubits. Although the coupling strengths between qubits are fixed, we effectively switch particle exchange off for state preparation and readout by detuning the qubits to different frequencies (inset in Fig. 2a). The common drive we use to generate the coherent-like states is applied to the system by means of the readout feedlines and couples to each qubit through the respective readout resonator of the qubit.

To measure site populations and correlators, we make single-shot measurements of identically prepared systems and then determine the expectation value of each operator as its average value across all measurements. To measure *X* and *Y* Pauli operators, we apply site-selective control pulses immediately before measurement.

For tomography measurements, we simultaneously measured 163 subsystems up to volume *V* = 6 by taking 2,000 single-shot measurement samples for each of the 3^6^ necessary Pauli strings, as visualized in Supplementary Fig. 14. The set of 3^6^ Pauli strings includes several copies of each of the 3^*V*^ Pauli strings needed to describe subsystems of volume *V* < 6. For subsystems of volume *V*, we can therefore extract 2,000 × 3^6−*V*^ measurement samples from our data. We extract density matrices using a standard maximum-likelihood estimator that is aware of individual single-shot outcomes. More detail is provided in the Supplementary Information.

### Entanglement across the particle-number manifolds

**Extended Data Fig. 1:**
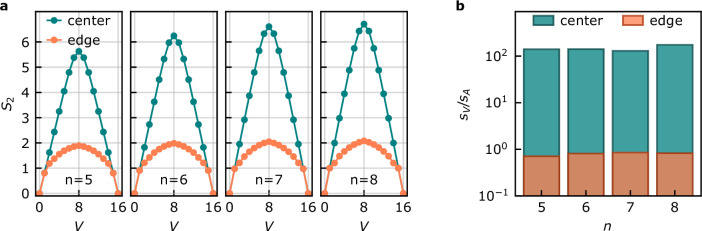
Entanglement scaling at different particle-number subspaces. **a**, Subsystem entropy versus volume of states at the centre and edge of the energy band for subspaces with *n* = 5, 6, 7 and 8 particles. **b**, Geometric entropy ratio *s*_V_/*s*_A_ comparison for different particle-number subspaces.

For each constant-particle-number manifold of the HCBH Hamiltonian, we observe a variation in the geometric entanglement from the edge to the centre of the spectrum^23^. To illustrate this variation, we report the average subsystem entropy as a function of volume for states at the edge and at the centre of the energy band of subspaces with *n* = 5, 6, 7 and 8 particles in Extended Data Fig. 1a. The states at the centre of the energy band exhibit a distinct Page curve, whereas the entropy of the states at the edge of the energy band shows a weak dependence on volume. Furthermore, in Extended Data Fig. 1b, we show the geometric entanglement ratio *s*_V_/*s*_A_ and notice the same trend between the states at the centre and at the edge of the energy band for the subspaces designated by the different number of particles. The geometric entanglement behaviour is consistent across different particle-number subspaces, allowing us to probe the entanglement scaling across the many-body spectrum using a superposition of different eigenstates.

### Measurement sampling statistics

Full-state tomography of a subsystem *X* containing *V*_*X*_ sites involves measurement of Pauli strings ${\prod }_{i\in X}{\sigma }_{i}^{{\alpha }_{i}}$ for all combinations of Pauli operators *α*_*i*_ ∈ {*x*, *y*, *z*}. For each Pauli string, we aim to accurately determine the distribution of measurement outcomes, of which there are ${2}^{{V}_{X}}$. For larger subsystems, the number of possible measurement outcomes is large, and as the state being measured approaches a volume-law state, the distribution of measurement outcomes approaches a uniform distribution. In this limit, the number of measurements required to accurately sample the outcome distribution becomes large.

The area-law states generated when |*δ*|/*J* is larger feature far-from-uniform distributions of measurement outcomes. Reconstruction of these states is therefore less sensitive to finite sampling statistics. This observation is commensurate with the results of ref. ^50^, in which only 3 × 10^3^ samples per Pauli string were sufficient to accurately reconstruct ten-qubit Greenberger–Horne–Zeilinger states (which have area-law entanglement scaling).

To quantify the impact of the number of samples *n*_s_ on the extracted entropy, we take a Monte Carlo approach. Here we consider the coherent-like state prepared at *δ* = 0 and Ω = *J*/2 (a volume-law state) and begin by obtaining the final state through a decoherence-free numerical simulation. For each subsystem and each Pauli string, we then sample from the distribution of bitstring measurement outcomes *n*_s_ times. We reconstruct the subsystem density matrices from these samples and compute their entropy *S*_2_. Density-matrix reconstruction used the same maximum-likelihood estimation routine as was used to reconstruct density matrices from experimental data.

**Extended Data Fig. 2:**
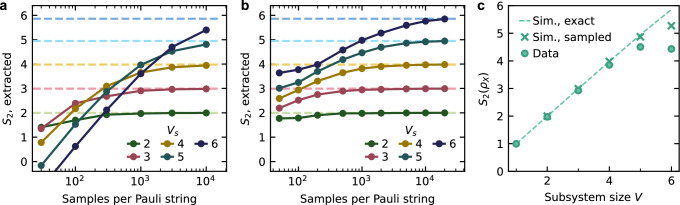
Simulation of the measurement sampling statistics problem. The dark markers present the second Rényi entropy extracted from simulated tomography of subsystems as a function of the number of measurement samples of each Pauli string used for density-matrix reconstruction. For each subsystem volume, the results were averaged over the same subsystems used in the experiment. The probability distribution of measurement outcomes was determined from the simulated state prepared at Ω = *J*/2 and *δ* = 0. The light dashed lines represent the exact entropy of the simulated state at each volume. **a**, Density matrices are reconstructed from the Stokes parameters obtained from Monte Carlo sampling of the probability distribution of eigenvalues of Pauli operator strings. **b**, Density matrices are reconstructed using maximum-likelihood estimation on the bitstrings obtained from Monte Carlo sampling of the probability distribution of measurement outcomes of each Pauli operator string. Maximum-likelihood estimation is not used in **a**, whereas the simulation in **b** uses the same reconstruction procedure that was used for experimental data. **c**, The entropy extracted from Monte Carlo sampling of the simulated state at Ω = *J*/2 and *δ* = 0 (crosses) is compared with the entropy extracted from experimental data (circles) (Fig. 3c). The values show the mean entropy of all subsystems at each volume. The exact entropy of the simulated state is shown for comparison (dashed line).

The results are shown in Extended Data Fig. 2 for *n*_s_ ranging from 50 up to 2 × 10^4^. Density-matrix reconstruction without maximum-likelihood estimation is shown for comparison. For low *n*_s_, sampling bias causes a biased reconstruction of the distribution of measurement outcomes, resulting in a deficit of the extracted entropy. The extracted entropy increases and eventually saturates at the correct values as *n*_s_ increases. The value of *n*_s_ needed to accurately extract the subsystem entropy grows exponentially in subsystem volume. Although *n*_s_ = 2 × 10^3^ was used for *V* = 6 subsystems in the present experiment, these simulations show that *n*_s_ ≳ 10^4^ is needed to accurately extract the entropy of volume-law states for subsystems of volume 6.

The results from the Monte Carlo simulation of measurement sampling effects are compared with experimental data in Extended Data Fig. 2c. Owing to the simultaneous tomography of all subsystems, our data yield 2,000 × 3^6−*V*^ measurement samples for a volume *V* subsystem.

## Online content

Any methods, additional references, Nature Portfolio reporting summaries, source data, extended data, supplementary information, acknowledgements, peer review information; details of author contributions and competing interests; and statements of data and code availability are available at 10.1038/s41586-024-07325-z.

### Supplementary information

Supplementary Information

## Data Availability

The data that support the findings of this study are available from the corresponding author on reasonable request and with the cognizance of our US Government sponsors who financed the work.

## Code availability

The code used for numerical simulations and data analyses is available from the corresponding author on reasonable request and with the cognizance of our US Government sponsors who financed the work.
